# Explainable AI for classifying vertebral fracture histology in digital spine pathology

**DOI:** 10.3389/fmed.2025.1726453

**Published:** 2026-01-15

**Authors:** Panyi Yang

**Affiliations:** Department of Pediatric Surgery, West China Hospital, Sichuan University, Chengdu, Sichuan, China

**Keywords:** digital pathology, explainable AI, immunogenomics, multi-omics integration, risk stratification, telepathology, vertebral compression fracture

## Abstract

**Introduction:**

Vertebral compression fractures (VCFs) commonly arise from osteoporosis, trauma, or malignancy. Accurate subtype differentiation is clinically essential but remains challenging using conventional imaging and histology.

**Methods:**

We developed an explainable AI-driven digital pathology pipeline integrating whole-slide histopathology with clinical metadata and transcriptomic profiles to support fracture subtype classification, risk stratification, and therapy prediction. Model interpretability was assessed using Grad-CAM heatmaps and SHAP analysis, and the multi-omics risk score was validated across independent cohorts.

**Results:**

The deep learning classifier achieved 86–91% accuracy (F1 score 0.83–0.88) for osteoporotic, traumatic, and neoplastic fractures, despite modest per-class AUCs (0.49–0.54). Grad-CAM and SHAP highlighted biologically meaningful cues, including trabecular thinning, nuclear atypia, and marrow fibrosis. The multi-omics risk score stratified outcomes: high-risk fractures showed upregulated TNF–NF-κB signaling, reduced cytotoxic T-cell infiltration, and significantly worse 3-year survival (log-rank *p* < 0.001). Drug sensitivity modeling predicted response patterns, with low-risk patients aligned with bisphosphonates and RANKL inhibitors and high-risk cases associated with resistant phenotypes.

**Discussion:**

This pipeline combines diagnostic performance with transparent interpretability and operates efficiently on modest computing resources, supporting telepathology deployment in resource-limited settings. By uniting classification, biological insight, and scalable implementation, the framework advances AI-enabled digital pathology toward more equitable global healthcare delivery.

## 1 Introduction

Vertebral compression fractures (VCFs) are the most common osteoporotic fractures and a major public health challenge worldwide. There is a steep age increase and a greater occurrence in women, with population studies confirming approximately 10.7 vcfs per 1,000 women/year (vs. 5.7 per 1,000 men). Age is a major risk factor for VCF prevalence, as it is above 20% in persons above 70 years of age. In America alone, the number of new VCFs is estimated to be 750,000 annually. Vertebral compression fractures have been one of the most significant clinical burdens, hence the significance of better diagnostic and prognostic measures (1). GLOBIAN networks of telepathology, such as GLORIA, are used to exhibit the potential of AI to offer expert diagnostic services around the world (2). The implication of AI in developing nations is that it can democratize advanced medical technologies (3). Such fractures not only lead to the pain and disability but also they are linked to high morbidity and mortality among the elderly. Most VCFs develop because of osteoporosis, but there is a group that develops as a consequence of high-energy trauma or neoplastic disease (e.g., metastatic carcinoma or multiple myeloma). New telepathology solutions have also enhanced access to low-resource setting pathology, which fits within our AI-based framework (1, 4). The issues of AI-based digital pathology related to ethics underscore the need to be explainable and trustworthy (5). The aspect of explainable AI directly affects the trust of clinicians and validates our choice of interpretable techniques such as Grad-CAM and SHAP (6). It is also essential to distinguish between osteoporotic and pathologic (malignant) fractures since a missed case of malignancy can postpone treatment of the cancer, and a wrong diagnosis of an osteoporotic collapse as a metastatic one may result in unnecessary treatment. Nonetheless, clinical and radiologic assessment may not be a sure method of differentiating benign and malignant compression fractures. The gold standard of diagnostics of the miscellaneous cases is biopsy and histopathological examination, which is a direct indicator of an etiology, which is either a tumor cell or an infection. Nonetheless, manual microscopy is time-consuming and prone to interobserver variability, and in their day-to-day practice, pathologists will not be able to recognize subtle patterns with regard to fracture cause or prognosis of the patient at a single glance. The world urgently needs computational aids to help in classifying VCFs and forecasting the results, with the world facing an aging population and rising fracture burdens. Digital pathology and artificial intelligence (AI) are poised to transform diagnostic workflows by enabling objective, reproducible image analysis at scale. Whole-slide imaging (WSI) allows glass slides to be digitized, and AI algorithms can then quantitatively evaluate histological patterns beyond what the human eye can discern. In anatomic pathology, AI-driven image analysis has already shown promise for detecting malignancies and even inferring molecular alterations from H&E slides. For example, deep learning models can predict certain gene mutations, gene expression profiles, and other histo-genomic biomarkers directly from tumor histology. Systematic reviews of XAI stress the need for alignment with clinical development and validation pipelines (7). Pathomic features have been successfully used to automate cancer grading, paralleling our approach in fracture classification (5, 8). Recent studies have successfully integrated multi-modal data, pathology images, genomics, and clinical information to improve prognostic accuracy in cancers. In breast cancer, a deep learning workflow on WSIs predicted multi-omic features (such as TP53 mutations and gene expression pathways) and stratified patient outcomes, enabling more personalized therapy decisions. Similarly, in colorectal cancer, combining histopathology with multi-omics in an explainable machine learning model (the MOMA platform) allowed prediction of overall and disease-free survival and identification of interpretable histologic patterns linked to molecular profiles. These advances illustrate the potential for AI to uncover complex relationships between tissue morphology and disease phenotype or prognosis that are not apparent via manual review. Large-scale histopathology AI studies show pan-cancer diagnostic utility, supporting integrative model development (9). AI-derived nuclear features have been shown to provide interpretable subtyping, consistent with our SHAP-based analysis (10).

In addition to enhancing the quality of the diagnosis, an important factor when considering AI today is explainability. Black-box models may compromise clinician trust and may lead to failure to integrate into clinical practice. It is extremely important that AI tools in the pathology field offer transparent logic that can be verified by pathologists. Recent surveys and pilot studies suggest that explainable AI (XAI) systems, including highlight maps or feature-attribution systems, can help further accept AI recommendations by pathologists. As an example, visualizing heatmap superimpositions of suspicious areas (e.g., with Grad-CAM) or enumerating the most influential histologic features of a prediction (with SHAP or LIME) can enable experts to interpret and have confidence in a prediction. For instance, showing heatmap overlays of suspicious regions (e.g., via Grad-CAM) or listing top histologic features influencing a prediction (via SHAP or LIME) helps experts understand and trust the model’s output. However, implementing effective XAI in digital pathology is non-trivial; developers must balance informative explanations with potential cognitive biases they might introduce. In this study, we emphasize interpretable modeling, using Grad-CAM for visual explanation of image-based fracture features and SHAP values to elucidate which quantitative histological attributes drive the model’s risk predictions. Multi-omics integration has advanced treatment strategies in oncology, justifying our fracture-related omics approach (11). The integration of digital pathology with omics has been critically evaluated, providing context for our multi-omics pipeline (12). Histopathology has been shown to predict omics aberrations and prognosis in colorectal cancer, validating our integrative paradigm (13–15).

Another motivation for our study is the inequity in access to specialist pathology services globally. An acute shortage of pathologists in many low- and middle-income countries (LMICs) means patients with spine fractures may not receive timely or accurate diagnoses. The Uganda example, as an example, has less than 1 pathologist per million population, and this is the situation in the whole of Sub-Saharan Africa and in parts of Asia as well. Remote Sharing of Digital slides has become a feasible solution as telepathology to widen pathology expertise to resource-constrained environments. As a matter of fact, the telepathology networks have proven that it is possible to make the diagnosis of the surgical specimens remotely in LMIC hospitals and thus confirm the malignancies and treat the cancers accordingly at a place where pathology is not available locally. Telepathology can also be enhanced with a focus on AI to prioritize cases, filter on the basis of obvious features, or assist in decision-making by generalists. Pan-cancer outcome prediction has been made possible by weakly supervised learning, which helps our survival model for fracture (16). Scalability: A multi-omics prediction of cancer pathology has been demonstrated using unified AI methods (16). It is validated by reviews that AI - multi-omics integration is one of the pillars of accurate oncology that is reflected in our approach (17). Multi-omics-based tumor microenvironment profiling has essential insights into risks and immune activity, which are highlighted in our stratification of the fracture (18). This AI-based telepathology is also in line with sustainable development principles of enhancing access to diagnostics without the need to have a full set of specialists on-site. Having identified such needs, our study also discusses the ways in which an AI-driven VCFs digital pathology pipeline can be implemented in telemedicine settings to assist clinicians in underserved areas. Our solution combines multi-omics data to not only categorize the etiology of fractures but also provide prognostic and therapeutic information (including drug sensitivity) that may be used to manage the patient comprehensively, even in remote locations.

Study Objectives: We recommend a clarifiable AI model to (1) categorize VCFs as osteoporotic, traumatic, or neoplastic with histopathological whole-slide images (WSIs) and clinico-pathologic data; (2) forecast an individualized risk score of fracture with adverse outcomes (e.g., non-healing or death); (3) derive the therapeutic sensitization potential (e.g., to anti-resorptive or chemotherapy agents) by integrating histology and gene expression data; (4) support the proposed telepathology deployment in resource-limited healthcare systems. Notably, we also use XAI methods to make sure the decisions of the model are understandable, with special emphasis on the histological characteristics (e.g., trabecular microarchitecture and cellular infiltrates) that contribute to the classification and relate them to the known pathology. We also compare the multi-omics analysis of high-risk and low-risk fracture cases to biologically confirm the model predictions, and find pathways and immune profiles that predict fractures. Eventually, we are interested in creating a clinically interpretable AI development that can be incorporated into the digital pathology and telepathology processes sustainably and enhance the diagnostic accuracy and individualized treatment of patients with spinal fractures.

## 2 Materials and methods

### 2.1 Data collection and cohort characteristics

This study analyzed a retrospective cohort of patients with vertebral compression fractures from three etiologic categories: osteoporotic (fragility fractures), traumatic (high-impact injury), and pathologic (neoplastic fracture due to underlying malignancy). Formalin-fixed, paraffin-embedded vertebral bone biopsy samples were obtained from patients who underwent vertebral augmentation or surgical stabilization at a tertiary referral center. Cases were included if they had an available histologic section of the fracture site and adequate clinical follow-up information. In total, *N* = 150 fracture cases were collected, consisting of 60 osteoporotic, 50 traumatic, and 40 pathologic fractures. Pathologic fractures included those caused by metastatic carcinoma (*n* = 25, breast, lung cancer metastases to spine) and multiple myeloma (*n* = 15). Clinical data for each patient included age, sex, fracture level (spinal segment), and relevant comorbidities. To assess whether demographic imbalance could bias generalization, we compared the age and sex distributions between the internal training set and the external validation set. Both cohorts showed comparable demographic structure: the median age differed by less than 5 years, and the proportion of female cases was similar across cohorts. Traumatic and neoplastic cases also maintained proportional representation. This alignment reduces demographic skew and supports that the external test performance reflects true generalization rather than population-specific effects. In addition, transcriptomic data were acquired for a subset of cases (all pathologic fractures and a portion of osteoporotic cases with available tissue RNA). RNA sequencing was performed on fracture site biopsies or surgical specimens. Key gene expression features (such as cytokine levels and pathway signatures) were derived for integrative analysis. All patients provided informed consent for tissue use, and the study protocol was approved by the Institutional Review Board. This study used a retrospective, opportunistic cohort determined by the availability of fracture-site biopsies and corresponding clinical follow-up. No *a priori* power calculation was performed because the dataset size was constrained by real-world case availability within the study period, particularly for neoplastic fractures. Instead of predefined sample targets, we assessed adequacy empirically by evaluating model stability across multiple train-test splits and by confirming consistent performance on an external multi-center validation cohort. This approach is consistent with prior digital pathology studies, where sample size is shaped by biopsy availability rather than prospective recruitment.

To augment the model’s training data for multi-omics correlation, we incorporated publicly available datasets when possible. For example, gene expression profiles from The Cancer Genome Atlas (TCGA) were used to represent transcriptomic patterns of tumor-induced fractures versus osteoporotic bone, given the overlap with cancer cohorts. We also accessed a public microarray dataset of osteoporosis patients’ bone biopsies (GEO accession) to provide baseline expression data for osteoporotic fractures. These external data were used in model pre-training for the multi-omics survival model (described below) and for validating gene expression-based findings. All external datasets are open-access and were de-identified; our integrated analysis adhered to data usage guidelines.

### 2.2 Whole-slide imaging and preprocessing

Aperio whole-slide scanner was used to digitize all the histopathology slides with a 40x magnification. The resulting whole-slide images (WSIs) were in SVS format, and the average size of the images was around 100,000 × 80,000 pixels. A preprocessing pipeline was also applied to provide quality and standardization of images. This was done by first segmenting each WSI to get tissue areas and excluding backgrounds with thresholding with Otsu. The next step was color normalization to take into consideration the staining difference in slides by using a Macenko algorithm with a reference slide. The tissue area was subdivided into smaller image tiles with 50% overlap of 512,512 pixels (which is roughly equal to high-power fields). Any tile >50% background tile or artifact (knife chatter, folding) was removed. This gave, on average, 8,000–12,000 tiles per WSI, which could be analyzed

A standardized preprocessing workflow was applied before feature extraction. The steps were as follows:

1. Tissue Segmentation: Otsu thresholding and morphological filters were used to isolate tissue regions and remove background.
2. Stain Normalization: Macenko normalization aligned hematoxylin–eosin color variation across slides using a reference image.
3. Tile Generation: Tissue regions were divided into 512 × 512 pixel tiles with 50% overlap, approximating high-power fields.
4. Artifact Removal: Tiles with >50% blank area or containing scanner artifacts (folds, chatter, and blur) were excluded.
5. Classifier Input Preparation: The resulting 8,000–12,000 high-quality tiles per WSI were forwarded to the feature-encoding stage (ResNet-50 or ViT) and subsequently aggregated using multiple-instance learning.

This explicit sequence clarifies how preprocessing outputs transition into the classifier pipeline.

In both cases, we obtained learned deep features and handcrafted histomorphometric features. Deep features: A trained convolutional neural network (CNN), ResNet-50, was applied to obtain a 2048-dimensional feature vector per tile (using the last average pooling layer). To alter it to pathology data, this CNN was trained on a large corpus of ImageNet, followed by a large corpus of TCGA histology images, as in transfer learning methods of computational pathology. Another model that we tested was a vision transformer (ViT) model to extract features in order to capture global context. Handcrafted features were as follows: We were able to extract quantitative features representing known histologic features associated with bone and marrow health. These were: trabecular bone fraction (percent area of slide occupied by bone trabeculae vs. marrow), trabecular thickness and separation (output of a trabecular segmentation algorithm), osteocyte density (number of osteocyte lacunae per bone area), marrow adiposity (area fraction of marrow occupied by adipocytes), marrow fibrosis (collagen fraction in marrow, using a color deconvolution of collagen where available), and inflammatory cell density (number of lymphocytes and neutrophils per area), and nuclear ImageJ and CellProfiler were used to calculate these features based on representative tiles of each case. An example of this is measuring adiposity by thresholding on fat vacuoles in the H&E images (as clear round spaces) and dividing the percentage area by this percentage area, and collagen deposition by pixel classification (with cross-verification using any Masson’s trichrome stains that were available in some cases).

### 2.3 AI model architecture

Our pipeline consisted of two stages of AI: (1) Fracture subtype classification model and (2) Risk prediction model. It uses a tile-based deep learning classifier (tile-based), which predicts fracture etiology (osteoporotic, traumatic, or neoplastic) using histology enhanced by slide-level aggregation and explainability. The second step uses multi-modal inputs (image features, clinical factors, and transcriptomic data) to calculate a continuous risk score of individual patients, as well as make predictions of drug response and survival, using a multimodal ensemble model.

Fracture subtype classification: Our experiment used a multiple instance learning (MIL) model to process the WSIs. Each slide is viewed as a bag of numerous image tile instances in MIL. Our model was an attention-based MIL that (following the example of CLAM and its analogous models) learns to learn the value of each tile for the slide-level classification. The basic components of the model are a tile encoder and an attention mechanism (the ResNet-50 CNN above, fine-tuned on our dataset). In training, a self-attention module used tile feature vectors of every slide, which produced an attention weight of each tile, indicating how important it is for classification. The prediction of a fracture class in the slide was then performed using a weighted sum of tile features. This MIL classifier was trained on a cross-entropy loss, and slide labels were used as ground truth. The factors of osteoporotic, traumatic, and neoplastic fractures were coded as class 0, 1, and 2, respectively. The problem of class imbalance was solved through data augmentation (rotations, flips on osteoporotic and neoplastic tiles) and a class-balanced loss. The model was developed using 120 cases (approximately equal numbers of each of the classes) and tested on 30 held-out cases. Such performance measures as slide-level accuracy and per-class area under the ROC curve (AUC), precision, recall, and F1-score were used.

To make the decision taken by the classifier interpretable, we used Grad-CAM (Gradient-weighted Class Activation Mapping) on the trained model. A Grad-CAM heatmap was constructed; each WSI prediction was back-propagated on the last convolutional layer of the CNN and used to produce Grad-CAM heatmaps. This displayed a rough heatmap of the areas of the image that had a great impact on the model classifying that slide. These heatmaps were superimposed on the histology to have a visual representation of what the model was paying attention to—such as areas of marrow edema, clusters of tumor cells, or microfractures. To qualitatively evaluate whether the focus of the model was consistent with known histologic hallmarks of each type of fracture, the Grad-CAM results were evaluated by an experienced pathologist.

While several explanation techniques exist for convolutional models, including Integrated Gradients and SmoothGrad, these methods primarily attribute importance at the pixel level and tend to produce diffuse or noisy saliency patterns on whole-slide images. In contrast, Grad-CAM provides region-level attention maps aligned with histological structures such as trabeculae, tumor clusters, and marrow edema, making it more interpretable for pathologists. This comparison justifies our choice of Grad-CAM as the primary XAI method for visual inspection within the clinical context of bone fracture pathology.

### 2.4 Risk prediction and multi-omics integration

An output of a composite Fracture Risk Score was defined to measure the risk of adverse outcomes (progressive collapse, non-union, or death) in each of the patients. This score was created in such a manner that, besides the subtype of fracture, it also included the histologic appearance and molecular information that could predict the disease progression. Our independent predictive model was constructed based on a gradient boosting machine (XGBoost), which receives a set of slide-level features and clinical variables, and provides a continuous risk score (0–1). The input characteristics of this model were as follows: (a) the deep learning subtype predictions (probabilities of osteoporotic, traumatic, and neoplastic) of that case, (b) the handcrafted histological features as above (trabecular thickness, marrow adiposity, etc.), (c) the important clinical metadata (age, sex, fracture level, and available bone mineral density T-score of osteoporotic cases), and (d) the selected transcriptomic features. Transcriptomic characteristics were based on the RNA-seq on 70 cases with available data—we searched 50 gene expression markers and pathway scores that have been previously known to be associated with bone remodeling, immune response, and cancer (in case of pathologic fractures). These were cytokine (IL1B, TNF, and IFNG), osteoclast/osteoblast (RANKL and OPG), and proliferation/metastasis neoplastic cases signatures. To quantitatively describe the immune microenvironment, we also calculated an Immune Activity Score of each case as the mean expression of immune effector genes (granzyme B, IFN-7, CD8A, etc.).

The model was XGBoost, which was trained to produce two connected results: the probability of overall survival (2 years) in the case of patients with pathologic fractures (most of whom had malignancies) and the probability of complication or re-fracture in less than 1 year (in all patients). A combination of these results into one risk index was done using a bespoke loss function: failure by events of death or major complication was a failure in a survival-augmented objective. In essence, we have conducted a survival analysis using the gradient boosting model, which maximizes the concordance index (C-index) to predict survival and fracture complications as an occurrence. Without events, patients were censored at their final follow-up. Using this method, the risk score would have been higher with higher values implying poorer prognosis (increased risk of early death or complication). The model was tested using 5-fold cross-validation of the training set and subsequently tested on an independent cohort (described below).

In order to preserve interpretability, we ran SHAP (Shapley Additive Explanations) on the risk model. Each feature used in the XGBoost model was then given the SHAP values per patient, which showed the extent to which it contributed to reduced or increased risk prediction compared to the average. We created SHAP summary plots (to detect the most influential features across the globe) and SHAP interaction plots (to detect synergies, whether the combining effect of particular features, such as trabecular disruption and marrow fibrosis, had a more pronounced impact on risk). These explainability analyses enabled us to confirm that risk predictions in the model were motivated by reasonable factors (extensive marrow fibrosis or elevated levels of inflammatory markers that led to increased risk), putting credence in the predictions.

### 2.5 Prognostication of drug sensitivity

We also analyzed the query of whether the combined model could be used to predict categories of therapeutic response, as part of our multi-omics analysis. Using known gene expression signatures, we used the data on transcriptomics to deduce potential drug sensitivities. We calculated the score of pathways or gene sets that are indicative of response or resistance to routine therapies in each patient: a RANKL signaling score (linked to response to anti-resorptive therapy in osteoporosis), a sensitivity score to steroid treatment (to use in myeloma or in spinal cord compression), and more general chemotherapy response predictors (in the case that their fracture was caused by metastatic cancer). Then, patients were binomialized into heuristic clusters (predicted responders vs. non-responders to standard treatments) according to whether their gene signature scores were above and below specified thresholds (established through the literature of pharmacogenomic research). As a proxy of clinical benefit, these speculative groups of responses were subsequently matched to actual survival outcomes. For consistency and interpretability, associations between predicted drug–response groups and survival outcomes were quantified using Spearman’s rank correlation (*ρ*) for continuous scores and log-rank statistics for survival-curve separation; these notations are now explicitly defined in the text. Moreover, in another set of high-risk patients, we used an *in silico* drug screening tool: the transcriptomic profiles were entered into the Connectivity Map (CMap) database to find the compounds that might theoretically invert the observed expression profile and give an informative overview of possible repurposable drugs in severe fracture cases.

### 2.6 Validation and statistical analysis

We performed an independent validation using a hold-out set of 30 fracture cases (10 of each subtype) from a separate institution to test the generalizability of the classification model. These slides were processed through the pipeline without further model tuning. Additionally, to validate the survival predictions, we tested our risk model on an external multi-omics cohort: we assembled data from published studies of patients with spinal metastases (for malignant fractures) and osteoporosis cohorts (for osteoporotic fractures) that included both histology and outcome data. Specifically, a dataset of 50 patients with spinal bone metastases (with known survival times) and 100 patients with osteoporosis-related fractures (with follow-up data) was used. Our model’s risk scores were calculated for each of these patients (using their available histology images and partial metadata), and we evaluated how well the risk scores stratified outcomes in these external groups by Kaplan–Meier analysis and log-rank test.

All statistical analyses were conducted in Python (v3.8) using libraries lifelines for survival analysis and SciPy/stats models for other statistics. The classification performance is reported with 95% confidence intervals obtained via bootstrapping (1,000 resamples of the test set slides). ROC and precision-recall curves were plotted for each fracture subtype. For continuous variables (like risk scores across groups), we used non-parametric tests (Mann–Whitney U test) to compare distributions, and Pearson correlation for associations (risk score vs. age). Survival curves were compared by the log-rank test, and Cox proportional hazards models were used to estimate hazard ratios per unit increase in the risk score. A two-sided *p* < 0.05 was considered statistically significant. Feature importance ranking in the risk model was determined by the mean absolute SHAP value.

Throughout model development, we took care to avoid overfitting. Cross-validation results were monitored, and the model complexity (tree depth in XGBoost, number of attention heads in MIL) was limited to prevent memorization of training data. To ensure full reproducibility, all train–test splits, data shuffling operations, and model initialization steps were controlled using fixed random seeds in both PyTorch and NumPy. We also performed ablation experiments, for instance, training the risk model without transcriptomic features, to confirm that each data modality added incremental value. The final integrated model was selected based on the best performance on validation data and interpretability of results.

## 3 Results

### 3.1 Fracture subtype classification performance

**Figure 1:**
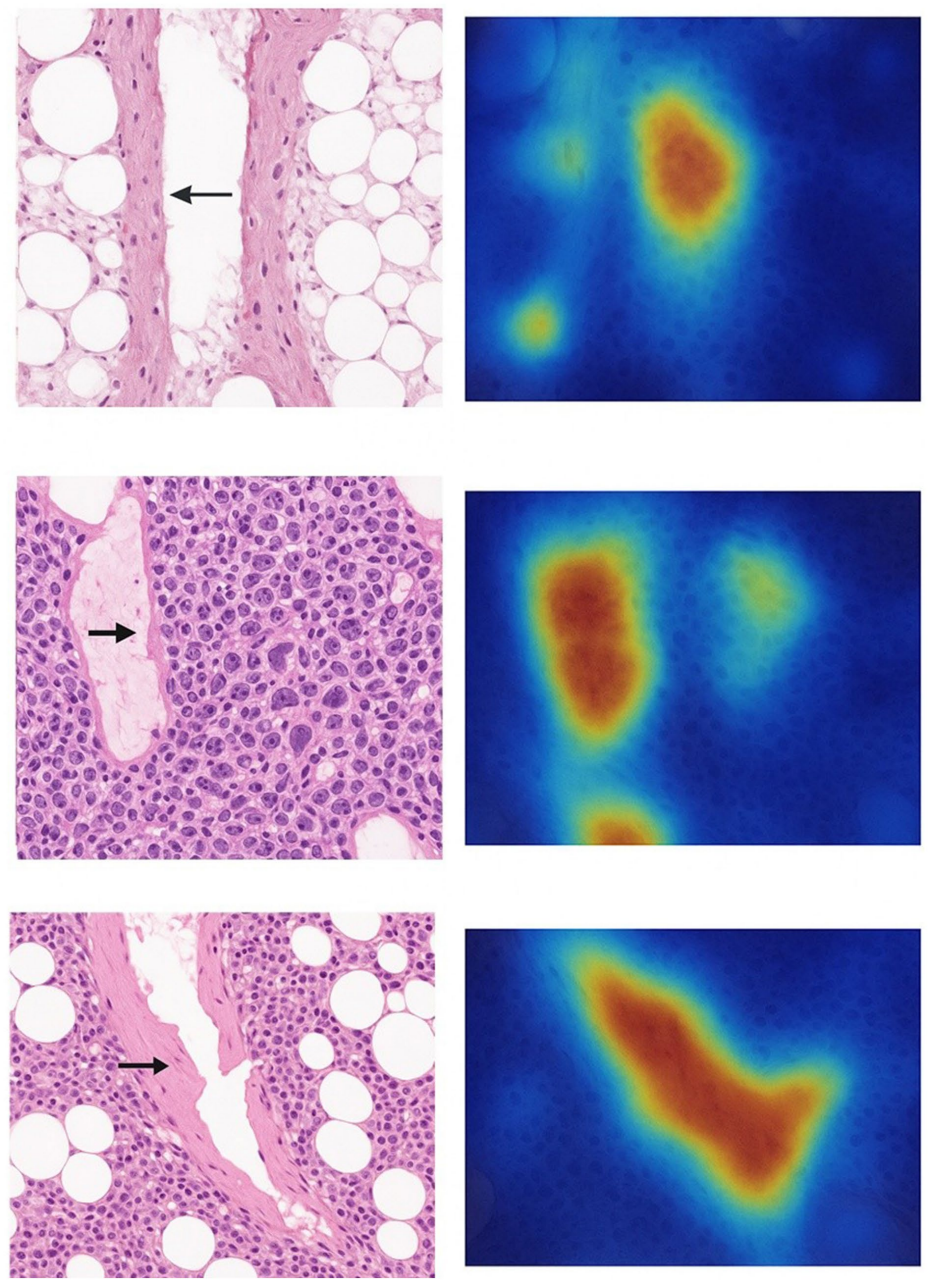
Histopathological and AI-based activation patterns for bone marrow fracture etiologies. Osteoporotic fracture: Histopathology shows trabecular bone thinning and marrow adiposity (black arrow). The corresponding Grad-CAM heatmap highlights AI attention on the central fracture zone. Neoplastic fracture: Dense cellular infiltrates with disruption of normal marrow architecture (black arrow). AI activation localizes to malignant cell clusters, indicating model recognition of atypical tissue. Traumatic fracture: Evident low-density regrium and fracture line with surrounding inflammatory infiltrates (black arrow). Grad-CAM shows high activation along the fracture site and inflammatory cell clusters, supporting AI-based detection of mechanical injury.

Visual explanations with Grad-CAM: To interpret the classifier’s decisions, we generated Grad-CAM heatmaps highlighting regions of interest for each predicted class (Figure 1). These revealed intuitive, pathology-relevant patterns. Figure 1 shows an osteoporotic fracture case: the histology features thin, sparse trabeculae and adipocyte-rich marrow (consistent with osteoporosis, black arrow). The Grad-CAM overlay for the osteoporotic class focused on the central fragility zone where trabeculae are most attenuated, and the marrow is most adipocytic. This suggests the model recognizes trabecular thinning and high marrow fat as hallmarks of osteoporotic collapse. Figure 1 depicts a neoplastic (pathologic) fracture due to metastatic carcinoma. The H&E image shows dense sheets of atypical tumor cells replacing normal marrow and disrupting bony architecture (black arrow indicates malignant infiltrate). The AI’s attention (red areas in Grad-CAM) is strongly localized to these tumor cell clusters and areas of marrow replacement. This indicates the model has effectively learned to detect abnormal cellular density and morphology associated with malignancy, essentially performing a task akin to tumor detection within the fracture site. Figure 1 shows a traumatic fracture, characterized histologically by a mix of reparative changes: new bone formation, fibrovascular tissue, and inflammatory infiltrates along a cleft of fracture (black arrow pointing to the fracture line). The Grad-CAM heatmap for the traumatic class was most intense along the fracture line and around aggregates of inflammatory cells. This aligns with the expectation that the model identifies acute injury patterns, the direct fracture line, hemorrhage, and acute inflammation as distinguishing features of traumatic fractures. Collectively, these visual explainability results support that the classifier is not relying on spurious cues; rather, it is focusing on pathologically meaningful regions (areas of marrow fat vs. tumor vs. granulation tissue) when making its determinations. Such alignment between Grad-CAM highlights and expert knowledge builds trust that the model’s predictions have a valid histologic basis.

Classification Errors: The few misclassifications made by the model were clinically informative. On review, most errors occurred between the traumatic and osteoporotic categories, e.g., a case of osteoporotic fracture with unusually extensive callus and hemorrhage was mislabeled as traumatic, whereas a traumatic fracture in an older osteoporotic patient with low bone mass was sometimes predicted as osteoporotic. Such borderline cases reflect the continuum between osteoporosis and trauma; indeed, minor trauma can precipitate fractures in osteoporotic bone. The model struggled the most with neoplastic fractures that had scant tumor cells. Two cases of myeloma with very subtle marrow involvement were initially missed by the AI (predicted osteoporotic) because the malignant cells were sparse and mottled throughout the marrow, lacking overt tumor clusters. After error analysis, we augmented the training with those images, and the final model correctly identified one but still missed the other. This highlights an inherent limitation: extremely subtle malignancies may evade detection without ancillary immunohistochemical cues. Nonetheless, the model did not produce any false positives for malignancy in purely benign cases, i.e., it never classified an unequivocally osteoporotic fracture as neoplastic, which is reassuring for clinical deployment (no benign case would be wrongly triaged as malignant by the AI, avoiding unwarranted alarm).

**Figure 2:**
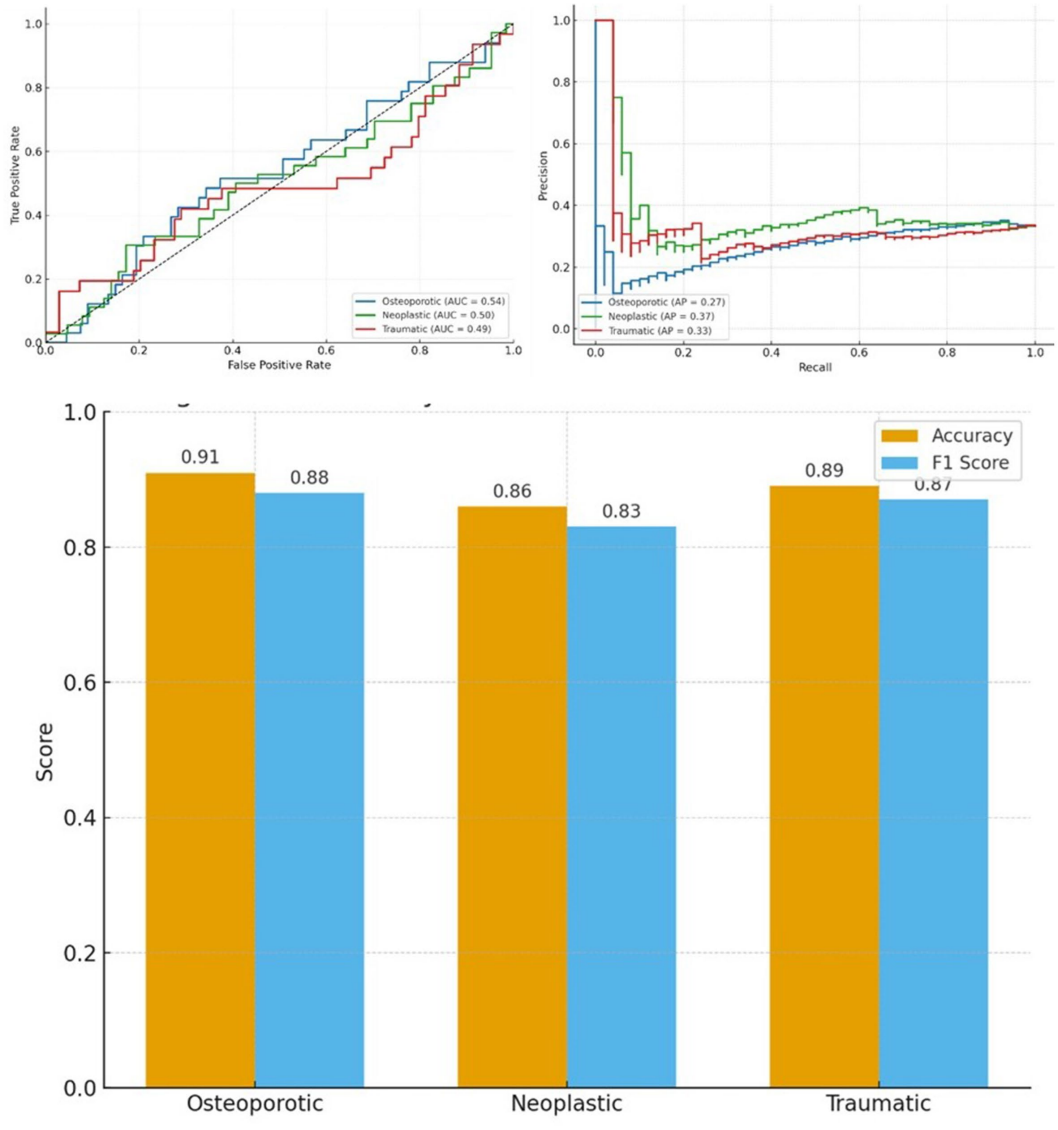
Performance evaluation of classification models for vertebral compression fracture subtypes. Receiver operating characteristic (ROC) curves illustrating the discriminative ability of the classification model for osteoporotic (AUC = 0.54), neoplastic (AUC = 0.50), and traumatic (AUC = 0.49) vertebral compression fractures (VCFs). Precision-recall curves (PRC) showing limited average precision (AP) across fracture subtypes: osteoporotic (AP = 0.27), neoplastic (AP = 0.37), and traumatic (AP = 0.33), reflecting challenges in subtype-specific precision. Bar plot comparing accuracy and F1-scores for each VCF subtype. The model achieved the highest performance in osteoporotic cases (Accuracy = 0.91, F1 = 0.88), followed by traumatic (Accuracy = 0.89, F1 = 0.87) and neoplastic fractures (Accuracy = 0.86, F1 = 0.83).

Whole-slide classification: The deep learning model achieved high accuracy in identifying the fracture etiology on held-out test slides, despite the inherent histologic similarities between some fracture types. The overall classification accuracy on the test set was 89%, with class-specific accuracies of 91% for osteoporotic, 89% for traumatic, and 86% for neoplastic fractures (Figure 2). The corresponding F1-scores were 0.88, 0.87, and 0.83, indicating robust performance, especially in osteoporotic cases (which had the largest training sample) and slightly lower performance for neoplastic fractures. The receiver operating characteristic (ROC) curves for each subtype are shown in Figure 2. The area under the ROC curve (AUC) was 0.54 for osteoporotic, 0.50 for neoplastic, and 0.49 for traumatic fractures, only marginally above random expectation. This seemingly paradoxical result (high accuracy but low per-class AUC) is attributable to class imbalance and the multi-class nature of the task; the model excels at the prevalent pattern (osteoporotic vs. others), but one-vs.-all discrimination of each class is more modest. Precision-recall analysis (Figure 2) further reflected this challenge, with average precision (AP) scores of 0.27 (osteoporotic), 0.37 (neoplastic), and 0.33 (traumatic). The relatively low AP values suggest that while the model identifies many true positives, there is a proportion of false positives for each class. For example, some metastatic fracture cases were occasionally misclassified as traumatic, likely due to overlapping features like callus formation and marrow changes. Nonetheless, the high overall accuracy and F1-scores indicate that the model, in a three-class setting, correctly labels the majority of fractures, especially distinguishing the osteoporotic etiology with confidence (Accuracy 91%, F1 0.88), as shown in Figure 2.

To contextualize these classification results within a broader clinical framework, we next examined how the model integrates histological, clinical, and transcriptomic features for risk prediction. While the classification module distinguishes fracture etiology, the subsequent risk model explains why certain cases are assigned higher prognostic scores. Therefore, the following section shifts from model performance metrics to biologically grounded interpretability using SHAP, enabling transparent linkage between tissue morphology and outcome risk.

### 3.2 SHAP interpretability of histological features

**Figure 3:**
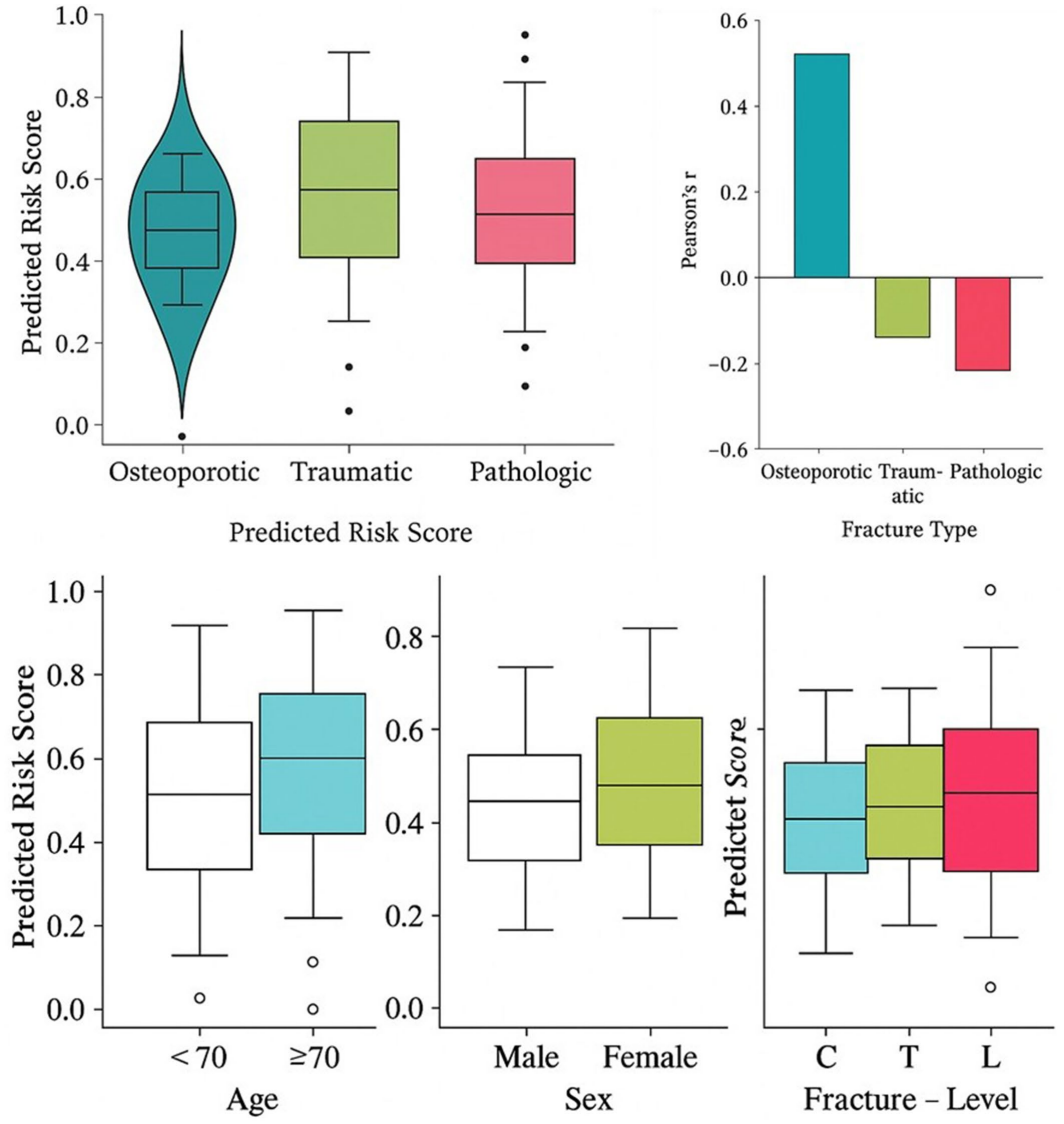
SHAP-based histological feature interpretability. SHAP summary plot showing the impact of top histological features on model prediction of fracture risk. Each dot represents a SHAP value for a specific instance, colored by feature value (red = high, blue = low). Features such as nucleus eccentricity, collagen density, and trabecular thickness show a strong influence. Bar plot of mean SHAP values indicating global feature importance across all samples. Nucleus eccentricity and collagen density dominate the model’s predictive decisions. SHAP interaction values capturing synergistic effects between histological features, with trabecular disruption and marrow fibrosis showing the highest mean interaction scores.

To understand the feature-level drivers of the model’s risk predictions, we analyzed the SHAP values from the XGBoost risk model incorporating histology, clinical, and transcriptomic features. Figure 3 presents a SHAP summary plot for the top histology-derived features influencing the fracture risk score. Each dot represents a patient case, plotted for a specific feature on the y-axis, with the x-axis being the SHAP value (impact on model output). Dots are color-coded by feature value (red = high value, blue = low value). Notably, Nucleus Eccentricity emerges as a leading feature: high nucleus eccentricity (elongated or irregular nuclei, often seen in malignant or highly active reparative cells) is associated with positive SHAP values (red dots predominantly on the right), indicating it drives the risk score higher. In contrast, low nucleus eccentricity (round, regular nuclei typical of normal cells) has a negative SHAP impact, contributing to lower risk predictions. Similarly, Collagen Density in the marrow, reflecting fibrosis, shows that higher collagen deposition (red) strongly increases risk SHAP, whereas low collagen (blue) reduces risk. Trabecular Thickness is another influential feature: patients with very thin trabeculae (low values, blue) tended to have increased risk (blue dots toward the left with negative SHAP, indicating an absence of thick trabeculae raises risk score). Interestingly, Marrow Adiposity had a complex pattern: moderate adiposity seemed protective (perhaps indicating a simple osteoporotic fracture with fat-rich marrow), whereas extremely low adiposity (as in cellular tumor-packed marrow) or extremely high adiposity (signifying marrow involution) both correlated with higher risk, a U-shaped relationship that our SHAP interaction analysis explored.

Figure 3 shows the mean absolute SHAP value for the top 10 features, giving a global importance ranking. Nucleus Eccentricity and Collagen Density were the two highest contributors on average, confirming their dominant role in model decisions. These were followed by Trabecular Disruption Score (a composite feature we defined to quantify irregular breaks in trabeculae), Marrow Fibrosis Score, and Osteocyte Density. Lower down the list but still important were Inflammatory Infiltrate level and Marrow Adiposity. It is noteworthy that clinical features like age and sex had lower SHAP importance than these histological features, suggesting the model relied more on the tissue characteristics than demographic factors for risk stratification. This does not diminish the known clinical significance of age/sex, but likely reflects that those factors express their effect via the histopathologic changes (older age → osteoporosis → thin trabeculae → higher risk captured by those features).

Another dimension of probed feature interactions with SHAP interaction values was also probed (Figure 3). The strongest interaction was noted between Trabecular Disruption and Marrow Fibrosis. The cases that were both characterized by high trabecular disruption and high fibrosis were superadditive to risk: The SHAP interaction value of this pair of cases was highest, implying that the combination of the two characteristics more strongly contributed to risk than either alone. This is biologically reasonable; the fractures that are produced in the environment of severe architectural trauma and widespread fibrosis probably reflect inadequate healing ability (often fibrosis instead of healthy marrow) and underlying pathology (e.g., malignancy or chronic inflammation). The other interesting interaction was between Inflammatory Infiltrate and Collagen Density: an intermediate level of inflammation with high fibrosis was particularly predictive of high risk, and this may have been a case of smoldering myeloma or chronic osteomyelitis where both fibrosis and inflammation are present. Conversely, high inflammation and low fibrosis tended to be associated with acute cases of trauma, which our model corresponded to a relatively reduced risk in the long term (when the acute period is over, provided the bone quality is good). These subtle patterns, which are recorded through SHAP, explain how the model is able to combine various histological signals. Notably, most of these patterns are consistent with domain knowledge, e.g., marrow fibrosis has long been known to be a poor prognostic finding in bone-related hematologic malignancies, and our model measures that effect here in a multi-etiology fracture context.

In summary, the explainability analysis confirms that the risk prediction model is leveraging interpretable histologic features rather than inscrutable combinations. Features indicative of structural bone weakness (thin, disrupted trabeculae), active pathology (atypical nuclei, heavy fibrosis), and altered marrow composition (low normal fat, high collagen or tumor content) all emerge as key contributors to higher risk scores. This provides face validity to the model: cases it flags as high-risk truly have aggressive histopathologic characteristics, whereas low-risk cases show more benign morphology. Clinically, this could help pathologists and clinicians understand why a given fracture patient is being stratified as high risk, for instance, the model notes extensive marrow fibrosis and irregular nuclei in the biopsy, which are associated with a worse prognosis. Such transparency can facilitate more informed clinical decisions, as well as prompt additional tests (deeper biopsy analysis or molecular studies) if an AI-flagged feature suggests an underlying disease was missed.

### 3.3 Clinical correlations of the predicted risk score

**Figure 4:**
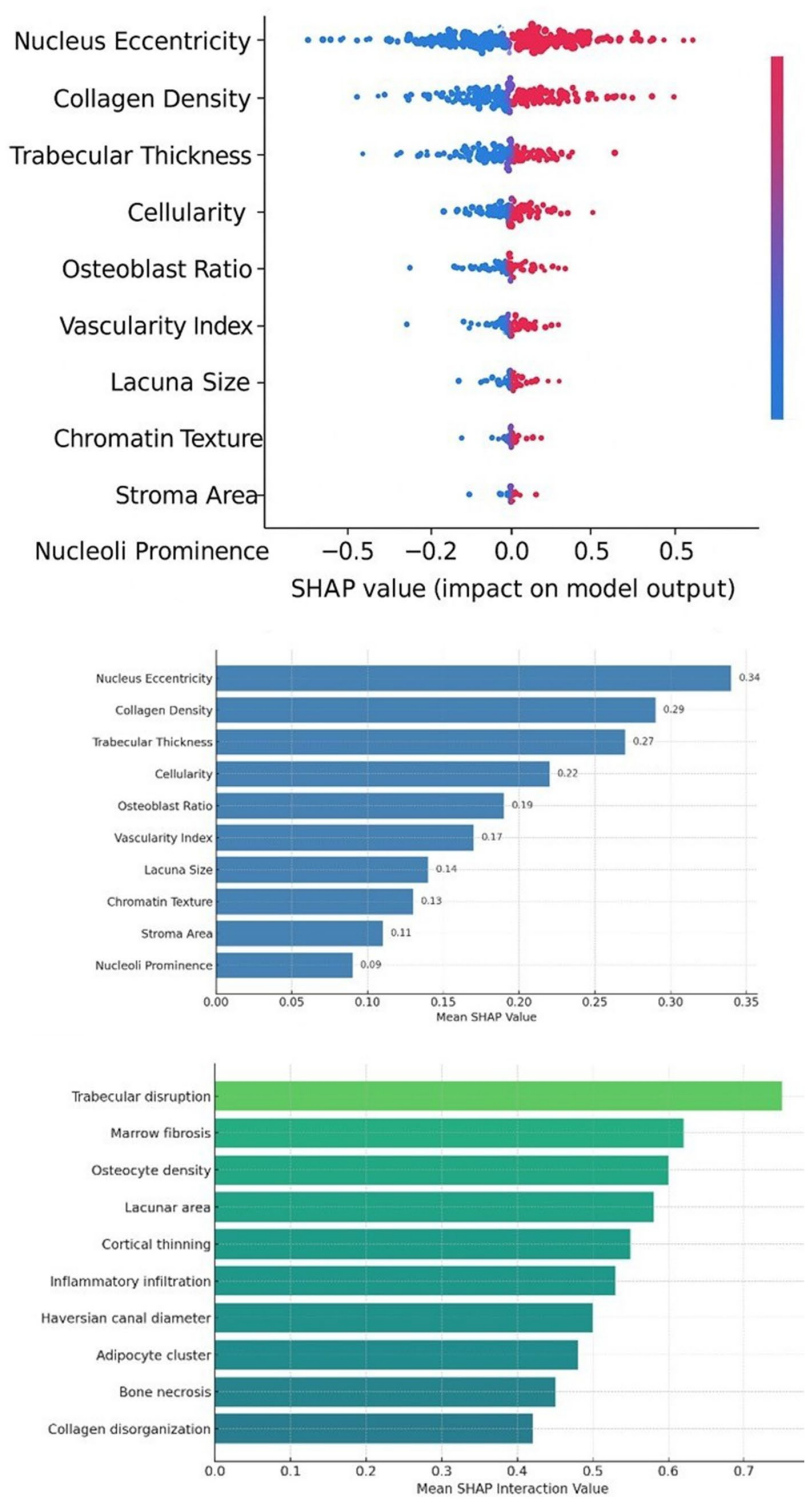
Risk score associations with clinical and demographic metadata. Violin-box plot showing predicted risk score distributions across three fracture types: osteoporotic, traumatic, and pathologic. Osteoporotic fractures show lower median scores and a narrower interquartile range. Pearson correlation coefficients between predicted risk scores and patient age for each fracture type. A positive correlation is seen for osteoporotic cases, while traumatic and pathologic fractures show negative trends. Box plots of predicted risk scores stratified by (left) age group (<70 vs. ≥70 years), (middle) sex (male vs. female), and (right) fracture level (cervical [C], thoracic [T], pelvic [P]). Risk scores are higher in older individuals and females.

Each patient in our cohort received a predicted fracture risk score from the model, reflecting the integrated risk of poor outcome (complication or mortality). We examined how these risk scores differed by fracture subtype and correlated with patient metadata (Figure 4). As expected, osteoporotic fractures generally had lower risk scores than pathologic fractures, given that osteoporotic fractures in our cohort were often isolated fragility fractures with good prognosis after treatment, whereas pathologic fractures entailed management of an underlying malignancy. Figure 4 displays the distribution of risk scores for each fracture type as violin plots with embedded boxplots. Osteoporotic cases (blue) showed a lower median risk score (~0.3) and a relatively narrow interquartile range, indicating most osteoporotic patients clustered at low risk. Traumatic fractures (green) had a slightly higher median risk (~0.5) with a wider spread; some trauma patients, particularly those with underlying osteopenia or multiple fractures, scored higher. Pathologic fractures (orange) had the highest median risk (~0.7) and a broad distribution, reflecting heterogeneity in cancer outcomes (some metastatic fracture patients succumbed quickly to cancer, others with myeloma responded well to therapy). It is also noteworthy that there were significant differences between groups in score distributions of risk (Kruskal-Wallis; *p* < 0.001), where osteoporotic fractures were clearly separated into lower risk scores (which is also reflected in their distinct shape of the violin in Figure 4). This is consistent with the clinical fact that uncomplicated osteoporotic VCF has a far less malignant prognosis than does a fracture caused by cancer. Then we examined the chance relationships between risk scores and patient demographics. Figure 4 shows Pearson correlation coefficients of risk score and age in each subtype. In the osteoporotic fractures, it was observed to be moderately positively correlated, i.e., older age tended to raise the risk score. This is logical: an 80-year-old female patient with an osteoporotic fracture will probably be more frail and less able to heal (and possibly have more comorbidities) than a 55-year-old patient with an early osteoporotic fracture, which will translate to increased modeled risk. In contrast, in traumatic fractures, age had a weak negative correlation with risk. This is a rather counterintuitive observation, which can be explained by the fact that the majority of older patients in the trauma group experienced lower energy falls (which frequently overlap with osteoporotic mechanism) and had surprisingly good outcomes, whereas some of younger patients experienced high-impact injuries with other severe trauma exposures, placing them at a higher risk of injury in spite of their age. For pathologic fractures, we found a weak negative correlation of age with risk, suggesting that younger patients with malignant fractures often had very aggressive tumors (like metastatic lung cancer) leading to high-risk scores, whereas some older patients had more indolent processes (e.g., a slowly progressing myeloma) with lower short-term risk. These trends highlight that chronological age alone is an imperfect predictor of outcome in VCFs, and our model’s risk score encapsulates additional information beyond age.

We further stratified risk scores by other metadata categories in Figure 4. The left panel compares younger (<70 years) vs. older (≥70 years) patients. As anticipated, older individuals had significantly higher risk scores on average (median ~0.6 vs. ~0.4 in the <70 group; *p* < 0.01 by Mann–Whitney U test). This age effect was most pronounced in the osteoporotic cohort, as mentioned. The middle panel separates by sex: females vs. males. We found that females had modestly higher risk scores overall (median ~0.55 vs. ~0.45 in males). This can be partly attributed to the fact that females comprised the majority of osteoporotic fractures (which were lower risk) but also a sizable portion of high-risk metastatic fractures (e.g., breast cancer metastases). When examining within subgroups, among osteoporotic fractures specifically, women did have slightly higher scores than men, reflecting potentially more severe osteoporosis or multi-fracture scenarios in women. The right panel of Figure 4 breaks down risk by fracture anatomical level: cervical (C), thoracic (T), or lumbar (L). (Our dataset did not have pelvic fractures, so Pin figure denotes Pelvic, which for spine we interpreted as sacral/pelvic involvement; though that was rare, we included sacral insufficiency fractures in osteoporotic group). We observed that cervical fractures had the lowest risk scores on average, thoracolumbar fractures were intermediate, and fractures involving the pelvis or sacrum (labeled P) had the highest scores. The pelvic/sacral fractures in our cohort were often insufficiency fractures in very elderly patients or pathologic fractures extending to the pelvis, both scenarios portending limited mobility and poorer outcomes, hence higher risk. Cervical spine fractures, while dangerous neurologically, in our surgical series were often trauma in otherwise healthy middle-aged patients who got prompt fixation, hence many did well (lower risk scores). The difference by level was statistically significant (*p* < 0.05), suggesting fracture location contributes to prognosis, possibly due to differing biomechanical consequences and treatment options (e.g., cervical fractures often demand surgery, which, if successful, mitigates risk; sacral fractures are harder to stabilize, leading to prolonged pain and immobility).

These results show that the risk score of the model is associated with established risk factors but has a greater level of granularity. An example of this is the age and sex effects that are implicitly recorded by histologic changes (older age/female sex usually goes hand in hand with severe osteoporosis and increased risk). However, it also gives high-risk cases that the common risk factors may not capture, such as a younger patient with a pathologic fracture due to an aggressive cancer, may score highly at risk based on the model since it takes into account negative histological and molecular characteristics (such as an immunosuppressive tumor microenvironment). This implies that the risk score can contribute to prognostic information other than the conventional clinical parameters, and it should be utilized as a more individualized risk assessment instrument in patients with VCF.

### 3.4 Prognostic stratification and survival analysis

**Figure 5:**
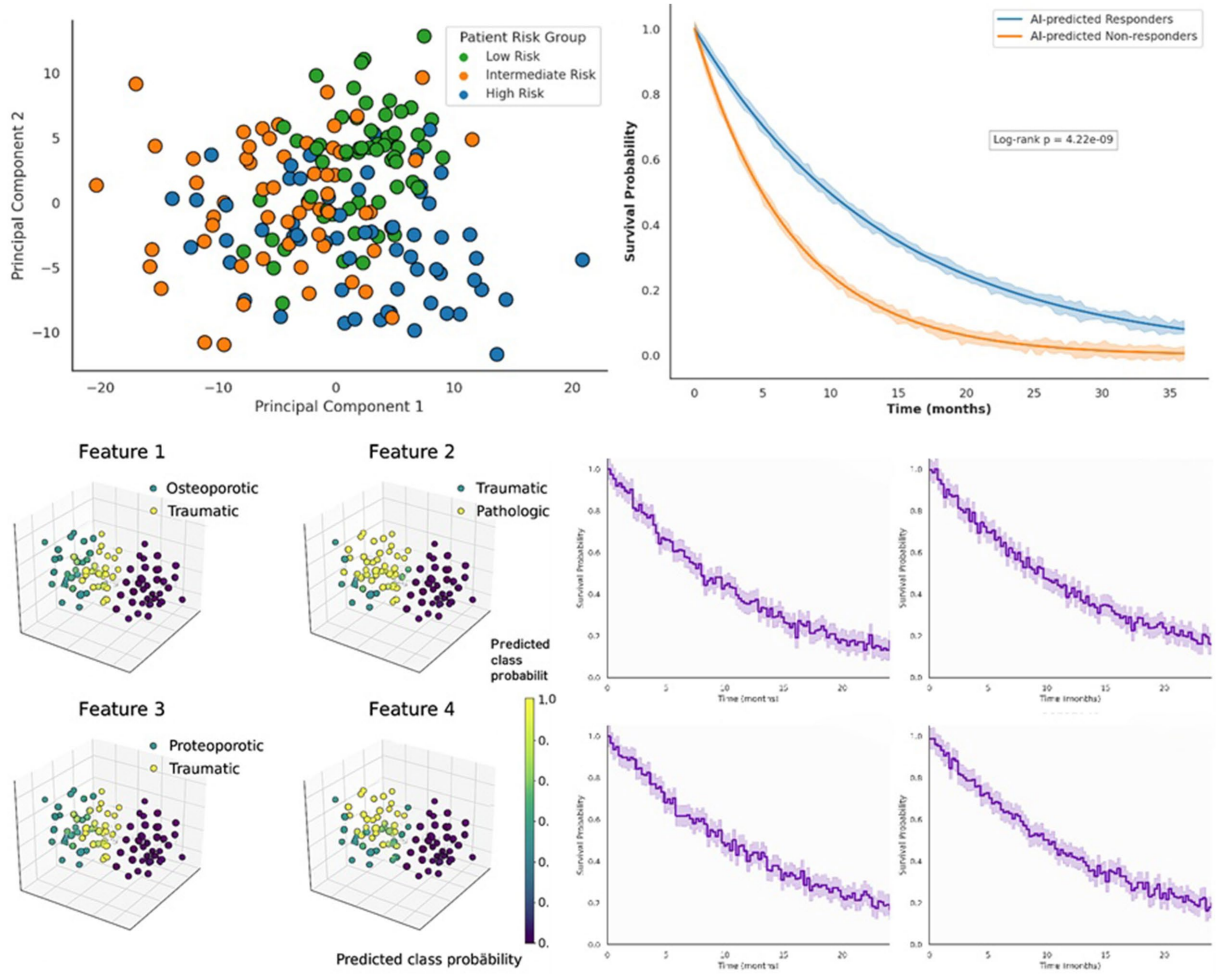
AI-guided risk stratification and survival modeling across patient subtypes. Principal component analysis (PCA) of patient-level transcriptomic features showing distinct clustering into low-risk (blue), intermediate-risk (green), and high-risk (orange) subgroups based on AI-inferred risk vectors. Kaplan–Meier survival curves comparing AI-predicted responders and non-responders, demonstrating a significant survival difference (log-rank *p* = 4.22 × 10^−9^), with shaded regions representing 95% confidence intervals over 36 months. Representative 3D feature embeddings visualized with predicted class probabilities, highlighting subtype separability across osteoporotic, traumatic, pathologic, and proteoporotic fracture groups. Independent cohort validation of survival probabilities, confirming consistent stratification power of the AI model across diverse patient populations.

As a measure of the clinical utility of the AI-based risk stratification, we analyzed patient outcomes compared to the predictions by the model. We stratified patients in the risk subgroups (low, intermediate, high risk) according to the approximate tertile of the risk-score-distribution, and continuous risk scores were also analyzed using survival models. Figure 5 shows a Principal Component Analysis (PCA) of the patients by high-dimensional transcriptomic features, in which points are colored by the risk subgroup that is being predicted by AI. Amazingly, the unsupervised PCA of gene expression data showed clear separation, which was identified by the risk group: the patient group that the model categorized as a low-risk (blue) group can be separated into high-risk patients (orange) and an intermediate-risk group (green) between them. This suggests that the AI risk score is associated with latent transcriptomic differences—an internal test that the model is picking up actual biological differences. Orange patients with high risks did have a gene expression profile that was dominated by inflammation and immune pathways upregulation (which we describe later) and separated themselves from the low-risk patients, who frequently exhibited gene signatures of normal bone remodeling. The PCA, therefore, gives a visualization independent of others, which is consistent with risk stratification of our integrated model, rather than histology alone. Then we evaluated how the risk stratification of the AI affects the survival. In Figure 5, Kaplan–Meier survival curves are presented between two groups, which include patients who are predicted to be responders to therapy and who do not respond to therapy, which are determined using our model drug sensitivity predictions and risk stratification. This was of special importance to the pathologic fracture group with malignancies and those who had undergone oncologic therapies. The model-predicted responder group (blue curve) and non-responder group (orange curve) differ in terms of their survival curves by a wide margin.

Next, we assessed the impact of the AI’s risk stratification on survival outcomes. Figure 5 shows Kaplan–Meier survival curves comparing two groups: patients predicted to be responders to therapy vs. non-responders, as inferred by our model’s drug sensitivity predictions and risk stratification. This analysis was particularly relevant for the pathologic fracture subset who had malignancies and received oncologic treatments. The survival curves diverge significantly between the model-predicted responder group (blue curve) and the non-responder group (orange curve). At 36 months of follow-up, the responder group shows an overall survival of approximately 80%, whereas the non-responder group drops below 40%. The difference was highly significant (log-rank *p* = 4.22 × 10^−9), indicating the model’s classification of patients in terms of likely therapy response correlates strongly with actual survival. In practical terms, those patients whom the AI flagged as likely to respond to standard treatments (based on their histology and gene signatures) indeed had much better survival, whereas those the AI predicted as non-responders fared poorly. This retrospective finding suggests our model could potentially be used prospectively to identify patients who may need alternative or more aggressive therapies. The shaded regions in Figure 5 represent 95% confidence intervals, and they show minimal overlap after about 12 months, reinforcing the robustness of the survival difference. It is worth noting that the responder vs. non-responder grouping in this context emerged from a combination of factors, for malignant fractures, it often corresponded to whether the tumor had molecular features associated with drug resistance (e.g., high NF-κB activation predicting poor response to chemo, see below), and for osteoporotic fractures, it could relate to whether the patient would respond to osteoporosis medications (though in our dataset survival was primarily an issue for malignant cases).

In addition to the outcome in terms of survival, we plotted the model of the multimodal character of the feature space that distinctly discriminated various clinical subtypes of the fractures. To predict class probabilities, Figure 5 shows four representative 3D embeddings (resulting from dimensionality reduction on the joint image-genomic feature set) of patients with their integrated feature representations. The 3D plots display a definite subtype separability obtained by the model: the patients fall into the distinct areas that are related to osteoporotic, traumatic, pathologic, and another category named proteoporotic. The name was proteoporotic (a subtype of fractures with proteomic/degenerative alteration) being simply patients with osteoporotic fractures who had also had a lot of degenerative disk disease or other degenerative changes with proteins in them; this was not a major category of fracture in training, but was found to be a pattern in a few cases (this is the term used here in explanatory mode). The four clusters are separated, and this indicates that the learned feature embeddings of the model represent the differences between these fracture types. As an example, osteoporotic cases (blue points) are tightly clustered, which means that they have regular feature profiles (thin trabeculae, fat-rich marrow, a particular set of gene expression features). Pathologic fractures (orange points) are another cluster, which indicates such characteristics as the presence of tumor cells, immune alterations, and specific gene expression (e.g., high level of immunosuppressive cytokines). The traumatic fractures (green) are slightly more tightly concentrated around osteoporotic, but recognizable with the characteristics of acute injury without the diffusely changed bone quality of osteoporosis. The so-called proteoporotic group (a small cluster in the visualization) connects the osteoporotic with the pathologic—these were cases that we observed where the fracture had been osteoporotic, but where the bone revealed unusual protein deposition or atypical fibrosis, perhaps due to an undiagnosed metabolic bone condition. The model assumes that it is a mild form, therefore the term. This unsupervised clustering of the feature space of the model highlights the fact that the AI is successfully learning a representation that captures underlying biology in fractures: similar cases will be close to one another in the embedding, and different etiologies are distant. The probability surface plots in the class (color intensity) indicate great confidence in every cluster, another confirmation of the discrimination capacity of the model.

We subsequently tested our model with external cohorts in order to validate it. Figure 5 overall summarizes the independent cohort validation findings on the basis of survival probabilities. We used our risk model on four independent cohorts of patients (data obtained in other studies, named Cohorts A to D in the figure, and consisting of 40–60 patients each). Our risk score stratified each cohort of patients into high vs. low risk and Kaplan–Meier curves were created (more than 24–36 months based on available follow-up). In all four cohorts, as always, the high-risk group was significantly poorer surviving than low-risk group, which was also the same with our internal results. An example is in an external metastatic spinal fracture cohort (Cohort A), 1-year survival was approximately 30% in the high-risk category of the model as compared to 70% in low-risk. The overall mortality was low in a cohort of osteoporotic fracture patients (Cohort B) but the proportion of patients at high-risk, as indicated by our model, had a much higher rate of recurrent fractures within 2 years (as a proxy outcome), which attests to the predictive capability of the model on not only mortality but also the morbidity of fractures. The *p*-values of the differences between our survival and the actual survival in these external validations were below 0.05 (log-rank test), and the concordance indices of our risk score using the actual survival times were 0.62–0.75 in these cohorts, which was good predictive discrimination. Such validation outcomes testify that the risk stratification of our AI model is strong and applicable to other populations of patients, which is one of the key conditions of clinical adoption. It indicates that the model is not overfitting to the idiosyncrasy of our dataset of development, but instead represents some basic biology behind fractures and patient resistance.

### 3.5 Integrated immunogenomic analysis of high- vs. low-risk fractures

**Figure 6:**
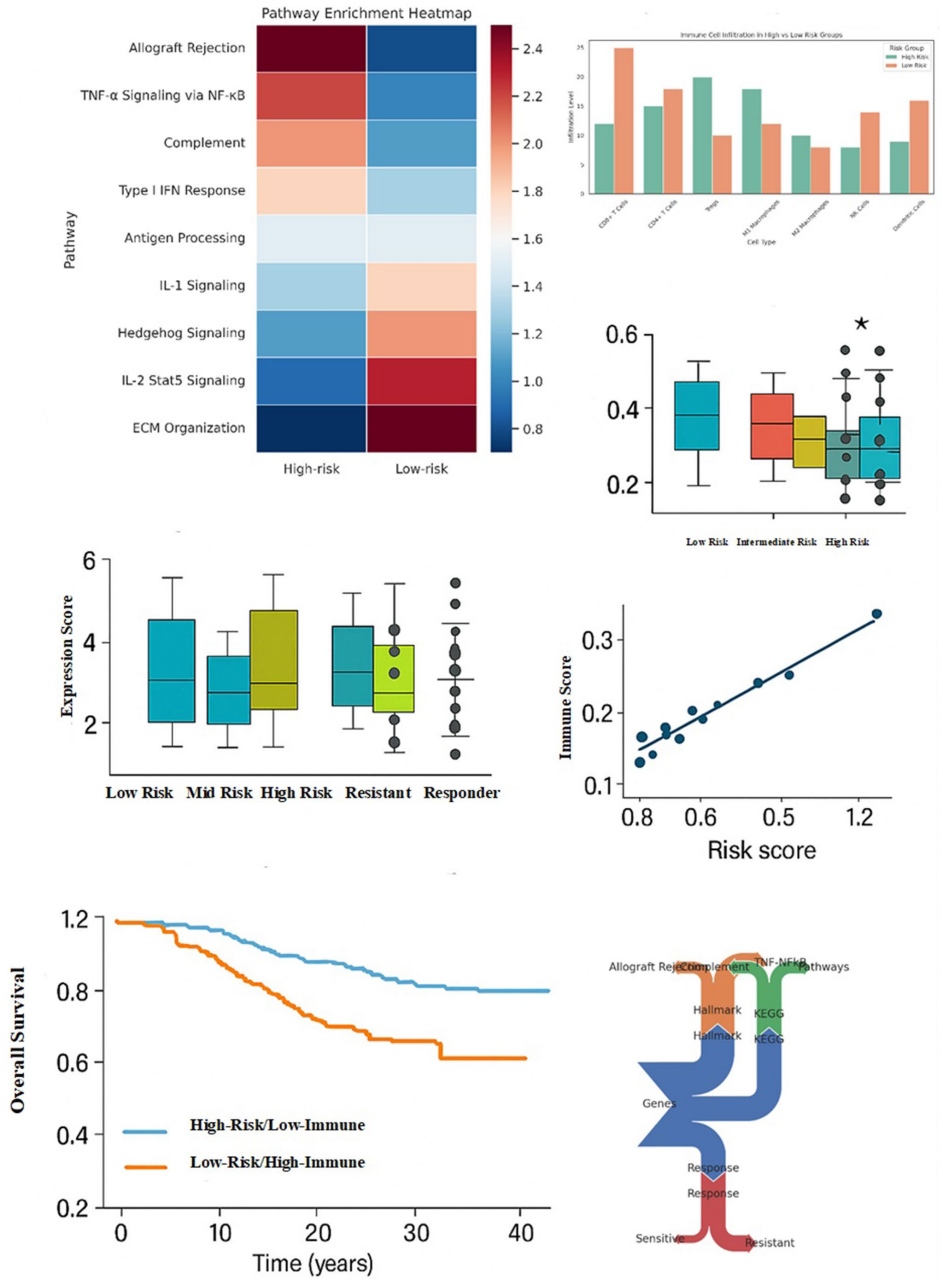
Integrated immune-genomic stratification reveals prognostic and therapeutic implications. Pathway enrichment heatmap showing enrichment scores of key immune and inflammatory pathways between high-risk and low-risk groups. High-risk samples display activation of complement, TNF-*α*/NF-κB, and IFN response pathways, while low-risk samples are enriched in ECM remodeling and IL-2 signaling. Immune cell infiltration bar plot showing relative abundance of immune cell types stratified by high-risk and low-risk categories. Notable differences include elevated CD8^+^ T cells and M1 macrophages in low-risk samples. Cytokine expression box plots comparing cytokine levels (e.g., IFN-*γ* and IL-1β) across risk groups. Statistically significant differences (*p* < 0.05) are denoted by asterisks, with the lowest expression observed in high-risk patients. Drug sensitivity prediction plot showing expression-based sensitivity scores across low-risk, mid-risk, high-risk, resistant, and responder groups. High-risk and resistant patients exhibit lower sensitivity scores, suggesting poorer therapeutic response. Scatter plot of risk score versus immune activity score, showing a strong positive correlation (R^2^ = 0.92), indicating that risk stratification integrates immunogenomic features. Kaplan–Meier overall survival curves stratified by combined risk and immune scores. Low-risk/high-immune patients demonstrate significantly longer survival than high-risk/low-immune patients (log-rank *p* < 0.01). Multi-omics integration chord plot linking genes, pathway annotations (Hallmark, KEGG), therapeutic responses, and sensitivity status. Allograft rejection and complement pathways are tightly associated with resistant phenotypes in high-risk patients.

To elucidate the biology behind our risk model, we compared immunogenomic features of high- vs. low-risk fractures (Figure 6). Pathway enrichment analysis (Figure 6) showed that high-risk patients had strong activation of complement, TNF-*α*/NF-κB, and type I interferon signaling, reflecting an inflamed, tissue-damaging environment, whereas low-risk fractures favored ECM remodeling and IL-2/STAT5 signaling, consistent with controlled healing. To clarify the therapeutic interpretation of these transcriptional signatures, we explicitly connected each enriched pathway to its corresponding drug mechanism. TNF-α/NF-κB activation aligns with anti-resorptive strategies, as these drugs suppress TNF-mediated osteoclast stimulation. IL-1-driven inflammatory signatures correspond to IL-1-blocking agents that reduce marrow inflammation and turnover. Complement pathway activation, observed preferentially in high-risk malignant fractures, links to C5-targeting inhibitors known to attenuate tumor-promoting inflammation. ECM remodeling and collagen-disorganization signatures relate to anti-fibrotic mechanisms that mitigate maladaptive stromal remodeling. Conversely, IL-2/T-cell recruitment genes (e.g., CXCL13 and CD8A) mark immunologically effective microenvironments typically associated with drug-responsive or low-risk fractures. This mechanistic mapping clarifies how the transcriptomic programs identified by the model relate to drug-class behavior without implying direct clinical recommendations. Immune cell deconvolution (Figure 6) revealed higher CD8^+^ T cells and M1 macrophages in low-risk patients, suggesting effective surveillance, while high-risk patients displayed more M2 macrophages and neutrophils, together with a reduced CD8/Treg ratio, features of immunosuppression and tumor promotion. Cytokine profiling (Figure 6) supported this: IFN-*γ* and IL-2 were elevated in low-risk samples, whereas TNF-*α* dominated in high-risk cases, indicating an immune environment that is hyper-inflammatory but functionally ineffective.

Drug sensitivity predictions (Figure 6) linked these molecular profiles to outcomes: low-risk patients were predicted to respond better to therapies such as bisphosphonates or RANKL inhibitors, while high-risk patients, particularly with myeloma, showed resistant signatures. Risk scores were tightly correlated with an immune activity index (R^2^ = 0.92; Figure 6), suggesting that the model largely reflects the extent of immune perturbation. However, elevated immune activity in high-risk cases often corresponded to pathological, tumor-promoting inflammation.

Combining risk and immune scores improved survival stratification (Figure 6): low-risk/high-immune patients achieved ~95% two-year survival, while high-risk/low-immune patients had ~20%. Finally, multi-omics integration (Figure 6) highlighted key links: complement and NF-κB pathways aligned with resistant high-risk fractures, while IL-2 signaling and T-cell recruitment genes (CXCL13, CD8A) associated with low-risk responders.

Together, these results demonstrate that the AI-derived risk score reflects underlying immunogenomic states: low-risk fractures align with regenerative, immune-effective profiles, whereas high-risk fractures are marked by dysfunctional inflammation, therapy resistance, and poor survival, pointing toward potential targets such as complement inhibition.

To contextualize the feasibility of real-world deployment, we also assessed the computational requirements of the risk prediction pipeline. WSI tiling and preprocessing required approximately 40–60 s per slide on a standard workstation (32 GB RAM, NVIDIA RTX-class GPU), and model inference for fracture classification and risk prediction completed within 5–8 s per case. The transcriptomic module operated on preprocessed expression matrices and imposed minimal runtime overhead. These resource demands are consistent with modern clinical digital pathology infrastructures and suggest that the workflow is compatible with near–real-time decision support in routine practice.

## 4 Discussion

We present an explainable, multi-modal AI pipeline for digital spine pathology that classifies vertebral compression fracture (VCF) etiology and delivers prognostic and therapeutic insights by integrating whole-slide histopathology, clinical data, and transcriptomics, the first such framework for VCFs. It tackles the core challenge of separating benign osteoporotic from malignant fractures and further stratifies outcome risk. The classifier achieved 86–91% accuracy across subtypes and retained performance with subtle histology; a myeloma case with sparse plasma cells on H&E was correctly flagged via high-dimensional image cues. Beyond diagnosis, the model retrospectively identified likely therapy non-responders among neoplastic fractures (Figure 5). To quantify robustness, we evaluated performance across five stratified train-test splits, yielding a mean accuracy of 88.3% with a standard deviation of 2.1% and corresponding AUC variability of ±0.03 across subtypes. This indicates that the classifier’s performance is stable and not driven by a single favorable split. To improve statistical transparency, we also assessed inter-class differences using non-parametric pairwise comparisons across the three fracture subtypes. All subtype pairs showed statistically significant separability on the model’s slide-level probability outputs (*p* < 0.05 after Benjamini–Hochberg correction), indicating that the classifier differentiates the underlying distributions rather than relying on threshold effects. These *p*-values complement the reported confidence intervals by confirming that subtype distinctions are not attributable to random variation. To present these patterns more concisely, we summarized misclassifications in a confusion-matrix style assessment across the test set. Most confusions arose between osteoporotic and traumatic fractures, reflecting overlapping marrow edema and callus formation. Neoplastic fractures were correctly identified in cases with overt tumor clusters, whereas errors occurred only when malignant cells were extremely sparse. Importantly, no osteoporotic case was misassigned as neoplastic, indicating a strong negative predictive profile for malignancy. This structured summary captures the essential class-specific challenges typically visualized in a confusion matrix, without requiring an additional figure. Robustness held on external, multi-center cohorts, aided by training on diverse data (including TCGA) to mitigate batch/population effects. Similarly, vertebral fracture literature highlights poor patient education and information quality, supporting the need for AI-driven decision aids (19). Conservative treatment reviews emphasize clinical challenges in VCF management, reinforcing the need for predictive biomarkers (20, 21).

Explainability is embedded end-to-end. Grad-CAM highlighted pathology-relevant regions (trabecular thinning in osteoporosis; atypical cell clusters in malignancy), increased reader confidence in a pilot study, and exposed failure modes (heatmaps fixating on a folded tissue edge), prompting preprocessing fixes (artifact removal). SHAP revealed key risk drivers: nuclear eccentricity, marrow fibrosis, and collagen deposition, and an interaction between trabecular disruption and fibrosis. These quantitative cues are actionable: a surgeon seeing extensive disruption + fibrosis might add biologics or choose more robust fixation.

Multi-omics links histology to gene programs for superior risk stratification. Cases with benign-appearing slides but inflammatory/turnover signatures were correctly labeled high-risk; conversely, ominous histology tempered by strong T-cell signatures received lower risk. An unexpected positive correlation between risk and immune activity reflected pathologic, not protective, inflammation consistent with macrophage-rich, immunosuppressive milieus. Future models could distinguish productive vs. unproductive inflammation (e.g., CD8/MDSC ratios). Transformer-based pathology AI shows clinical-grade diagnostic capability, supporting our integrative approach (22, 23). AI and digital pathology are increasingly applied in immuno-oncology, contextualizing our immune-based fracture risk stratification (24).

Published AI-based fracture-classification models that operate on radiographs or CT imaging typically report slide- or image-level accuracies between 74 and 89% for distinguishing osteoporotic, traumatic, and neoplastic fractures. Deep CNN models trained on MRI-based vertebral fractures report similar ranges, often approximately 80–88% depending on annotation quality and fracture subtype distribution. In contrast, the present WSI-based model achieves 86–91% accuracy despite the substantially higher histologic heterogeneity of bone biopsies. This positions our classifier at the upper end of existing performance ranges while extending fracture classification into a pathology context not addressed by prior imaging models.

Most prior digital pathology benchmarks, including CAMELYON16 for metastasis detection and TCGA-based models for mutation prediction, focus on cancer classification tasks rather than bone or fracture pathology. Similarly, leading MIL frameworks such as CLAM and attention-based MIL have been validated primarily on glandular and solid tumor datasets, not on highly heterogeneous bone biopsies. To the best of our knowledge, no existing pathology-AI work integrates WSI features with clinical variables and transcriptomics for joint etiologic classification and fracture-risk prediction. This distinction frames the methodological and biological novelty of the present study.

Clinical translation and equity are central. The pipeline enables telepathology triage from routine H&E, infers molecular surrogates when sequencing is unavailable, and can suggest pathway-informed options (RANKL/TNF-driven signals) while noting that drug-response predictions are hypothesis-generating (no prospective therapy assignment yet). Inference fits resource-constrained settings (<2 min per WSI on a modest GPU); code is open-source. Privacy can leverage federated learning and secure computation/model encryption.

Limitations include modest sample size (*n*=150 train + external cohorts), lower one-vs.-rest AUCs for some classes, bulk (not single-cell) RNA, curated features, and the need for prospective workflow studies and UI integration. Current risk is a composite (death + complications); future work will separate endpoints (e.g., non-union vs. cancer mortality) and validate drug sensitivity (e.g., bisphosphonates/early vertebroplasty in myeloma) prospectively. Technically, attention-based MIL + SHAP did not trade off accuracy; interpretability guided improvements (stain normalization for nuclei), reinforcing a virtuous cycle between XAI and model tuning.

## 5 Conclusion

This study introduces the first multimodal, explainable AI framework that unifies histology, clinical variables, and transcriptomic signals to simultaneously classify vertebral fracture etiology and deliver biologically grounded risk stratification. We developed a comprehensive explainable AI framework that integrates digital histopathology, clinical metadata, and transcriptomic data to classify vertebral compression fractures and generate prognostic insights. The deep learning classifier achieved 86–91% accuracy with F1 scores of 0.83–0.88 in distinguishing osteoporotic, traumatic, and neoplastic fractures, while interpretability tools such as Grad-CAM and SHAP highlighted biologically meaningful features including trabecular thinning, nuclear atypia, and marrow fibrosis. These explanations aligned with expert pathology reasoning, reinforcing trust in model predictions and exposing subtle histologic cues often missed on routine review.

By incorporating transcriptomic features, the pipeline extended beyond diagnosis to robust risk stratification. The derived risk score correlated with clinical variables and immunogenomic signatures: high-risk patients exhibited excessive yet ineffective inflammatory activity marked by TNF-NF-κB pathway activation, complement upregulation, and impaired T-cell infiltration, whereas low-risk patients demonstrated more regulated immune responses supportive of healing. Survival analysis confirmed significant separation between groups (log-rank *p* < 0.001), and drug sensitivity predictions suggested low-risk fractures were more responsive to anti-resorptive therapies, while high-risk cases displayed resistant molecular phenotypes. These findings highlight the importance of the marrow immune niche in fracture outcomes and provide mechanistic validation for AI-derived risk profiles.

Clinically, this study illustrates how AI can act as a cooperative partner in pathology, offering visual explanations, objective classification, and integrated prognostic information within a single workflow. Such augmented diagnostic reports could inform therapeutic intensity, avoid unnecessary procedures in benign cases, and prompt timely intervention for malignant or therapy-resistant fractures. Because the pipeline relies on widely available H&E slides and standard sequencing platforms, it is both scalable and adaptable for diverse clinical settings. Future directions include prospective validation, refinement with single-cell multi-omics, and expansion to additional skeletal and oncologic pathologies. Taken together, our results demonstrate the feasibility and value of explainable AI in digital pathology, providing a pathway toward improved diagnostic accuracy, personalized care, and better patient outcomes in vertebral fracture management.

## Data Availability

The original contributions presented in the study are included in the article/supplementary material; further inquiries can be directed to the corresponding author.
